# The Interplay between Oxidative Stress, Inflammation and Angiogenesis in Bladder Cancer Development

**DOI:** 10.3390/ijms22094483

**Published:** 2021-04-25

**Authors:** Paulina Wigner, Radosław Grębowski, Michał Bijak, Joanna Saluk-Bijak, Janusz Szemraj

**Affiliations:** 1 Department of General Biochemistry, Faculty of Biology and Environmental Protection, University of Lodz, 90-236 Lodz, Poland; joanna.saluk@biol.uni.lodz.pl; 2 Department of Urology, Provincial Integrated Hospital in Plock, 09-400 Plock, Poland; radek.grebowski@gmail.com; 3 Department of Medical Biochemistry, Medical University of Lodz, 92-216 Lodz, Poland; janusz.szemraj@umed.lodz.pl; 4 Biohazard Prevention Centre, Faculty of Biology and Environmental Protection, University of Lodz, 90-236 Lodz, Poland; michal.bijak@biol.uni.lodz.pl

**Keywords:** oxidative stress, inflammation, angiogenesis, bladder cancer

## Abstract

In 2018, 550,000 people were diagnosed with bladder cancer (BC), of which nearly 200,000 people died. Moreover, men are 4 times more likely than women to be diagnosed with BC. The risk factors include exposure to environmental and occupational chemicals, especially tobacco smoke, benzidine and genetic factors. Despite numerous studies, the molecular basis of BC development remains unclear. A growing body of evidence suggests that inflammation, oxidant-antioxidant imbalance and angiogenesis disorders may play a significant role in the development and progression of bladder cancer. The patients with bladder cancer were characterised by an increased level of reactive oxygen species (ROS), the products of lipid peroxidation, proinflammatory cytokines and proangiogenic factors as compared to controls. Furthermore, it was shown that polymorphisms localised in genes associated with these pathways may modulate the risk of BC. Interestingly, ROS overproduction may induce the production of proinflammatory cytokines, which finally activated angiogenesis. Moreover, the available literature shows that both inflammation and oxidative stress may lead to activation of angiogenesis and tumour progression in BC patients.

## 1. Introduction

Bladder cancer (BC), also known as urothelial cancer or urinary bladder cancer, is ranked 7th on the list of the most common cancers in men and the 17th in women worldwide. Unfortunately, the number of new cases continues to increase, especially in developed countries. In the Western World, the BC is the fourth most common cancer in men and the ninth in women [1]. It was estimated that 550,000 people were diagnosed with bladder cancer in 2018, which accounts for 3% of all new cancer diagnoses. Interestingly, the highest bladder cancer rates were noted in Southern, Western Europe and North America [2]. Bladder cancer is the 13th most deadly. In 2008, nearly 200,000 people died of BC, which accounts for 2.1% of all cancer deaths. However, the 5-year survival rate for bladder cancer is above 75% [3]. Additionally, men are 4 times more likely than women to be diagnosed with BC. The development of BC may result from exposure to environmental and occupational chemicals, especially tobacco smoke [3]. Tobacco is a strong carcinogenic agent because it is a rich source of polycyclic aromatic hydrocarbons, aromatic amines and N-nitroso compounds, which cause DNA damage via adduct formation, single- and double-strand DNA breaks and modifications of the base [4]. Thus, smoking tobacco increases the risk of developing bladder cancer even three times compared to never-smoking patients. Moreover, smoking causes about half of all bladder cancers [5]. Additionally, the previous analysis showed that the risk of BC increased with the duration of smoking (number of years smoked) and intensity of smoking (number of cigarettes smoked per day) [6,7]. In addition to smoking, risk factors of BC development are industrial chemicals, including aromatic amines (benzidine and 2-naphthylamine) used in the dye industry by makers of rubber, leather, textiles and paint products as well as printing companies [8,9]. Interestingly, already in 1954, study confirmed that exposure to 2-naphthylamine was associated with a 200-fold increased BC risk for English and Welsh workers in the rubber industry [10]. In industrial facilities in Leverkusen, Germany, 92 of 331 (27.8%) workers who had been exposed to benzidine had BC [11]. Moreover, a Chinese study showed that exposure to benzidine caused a 35-fold increase in BC risk [12]. The subsequent chemical compound associated with BC development is arsenic in drinking water. The countries characterised by a high concentration of arsenic in groundwater and surface soil are Bangladesh, China, Hungary, and India [13]. Previous studies showed that exposure to arsenic in drinking water at concentrations exceeding 300–500 µg/L is associated with BC occurrence [14,15]. Interestingly, another risk factor of BC is daily fluid consumption. People who drink a lot of fluids each day, especially water, tend to have lower rates of bladder cancer (1.4 L/day compared with 0.4 L/day) [16,17,18]. On the other hand, a meta-analysis confirmed that the high daily fluid intake might be associated with high BC risk in European and American males, and a limitation of fluid consumption to <2000 mL and <3000 mL per day is recommended, respectively [19]. Bladder cancer occurrence is also associated with age—the median age at diagnosis is 69 years for men and 71 years for women [20]. Thus, prolonged exposure to harmful factors in the elderly contributes to a greater frequency of this cancer in people over the age of 55. Due to the ageing population, the number of diagnosed cases of BC is increasing annually [21]. It is estimated that by the year 2030, around 219,000 people will be diagnosed across Europe each year [22,23]. By comparison, the overall number of estimated new bladder cancer cases was about 141,000 in the European Union in 2020 [24]. Interestingly, previous studies confirmed racial differences in BC incidence and mortality rates. On the one hand, white populations get sick twice as often as black populations. However, rates of mortality are higher among black populations than among white populations [25,26,27,28,29,30,31].

In addition to environmental factors, genetic factors influence the development of BC. First-degree relatives of bladder cancer patients were characterised by a twofold increased risk of this cancer development [32,33]. Previous studies confirmed that single nucleotide polymorphisms (SNPs) might modulate the risk of bladder cancer development. Validated genetic loci for BC risk include 8p22 (*NAT2*, *GSTM1*), 8q24.21 (*MYC*), 3q28 (*TP63*), 8q24.3 (*PSCA*), 5p15.33 (*CLPTM1L-TERT*), 4p16.3 (*TACC3-FGFR3*), 22q13.1 (*APOBEC3A-CBX6*), 19q12 (*CCNE1*) and 2q37.1 (*UGT1A*) [34]. Among the many polymorphisms, the SNPs localised in genes encoding acetyltransferase 2 (*NAT2*) slow acetylator and glutathione S-transferase μ1 (*GSTM1*) were characterised by relatively strong associations with the risk of BC. The slow acetylator genotype of *NAT2* and *GSTM1*-null genotypes were associated with increased BC risk [35,36]. NATs catalyse the acetylation of aromatic and heterocyclic amine carcinogens and drugs, leading to their activation or deactivation. Amongst them, two major isoforms can be distinguished in humans, NAT1 and NAT2. The N-acetylation of the compounds usually leads to detoxification, while O-acetylation causes activation of carcinogens [37,38]. In consequence, the changes of *NAT* genes might be associated with the risk of bladder cancer. The previous meta-analysis confirmed that the *NAT2* slow acetylator genotype was associated with a 40% increased risk of bladder cancer development. Interestingly, the correlation was stronger in the cigarette smoker group than the never-smoker group [35]. Similarly, Rothman et al. (2010) confirmed that polymorphism in the *NAT2* gene may modulate BC risk [39]. The G allele of *NAT2* polymorphism (rs1495741) decreased the risk of BC occurrence, while the A/A genotype associated with slow acetylation increased the risk by about 15%. However, the analysis of cigarette smokers and never smokers showed that the A/A genotype increased the risk in only cigarette smokers. The subsequent enzyme associated with BC development is GSTM1. GSTM1 takes part in the II phase of detoxification of carcinogens, including polycyclic aromatic hydrocarbons. The previous meta-analysis showed that *GSTM1*-null genotypes increased BC risk by about 50% [35,39]. Interestingly, the effect was weaker in the cigarette-smoking group. Never smokers with the *GSTM1*-null genotype were characterised by a 71% increased BC risk, former smokers by about 62% and current smokers by about 19% [39]. GWAS European selected other polymorphisms associated with the development of BC and are presented in Table 1. Previous studies confirmed that the mutations localised in *FGFR3*, *RB1*, *HARS*, *TP53* and *TSC1* genes might be associated with BC. The genes regulate the cell division preventing cells from dividing too fast and uncontrolled [40]. Moreover, mutations of *TP53* may be associated with the invasive character of bladder cancer [41]. Besides, *TERT* gene mutations have been confirmed in 70% of patients with BC. *TERT* encodes reverse transcriptase of the telomerase complex, which is involved in protecting DNA by increasing the length of telomeres located at the chromosome ends [42]. The subsequent gene associated with BC development is *p73*. The gene encodes a protein, which is involved in regulating the cell cycle, activation of apoptosis and cell differentiation and development. Patients with BC were characterised by overexpression of *p73*, which may be associated with the disease’s progression [43]. The missense mutation of *HRAS*, causing the replacement of the glycine with the valine at position 12, is also associated with BC. In consequence of mutations, the protein is activated and then causes growth of the cells and divides in the absence of outside signals, which leads to uncontrolled cell division and the formation of a tumour in the bladder. Moreover, the mutations of *HRAS* may be associated with the cancer progression and may also increase the risk of tumour recurrence after the anti-cancer treatment [41,44]. One of the most commonly mutated genes in bladder cancer is *STAG2*, which encodes the cohesin subunit STAG2. The protein plays a crucial role in the regulation of sister chromatid cohesion and segregation. The mutations of *STAG2* were observed in about 36% of patients with papillary non-invasive urothelial carcinomas and in 16% of invasive urothelial carcinomas of the bladder [45].

Despite extensive studies into the pathogenesis of bladder cancer, its molecular basis remains unclear. However, a growing body of evidence suggests that interrelated biochemical pathways, including inflammation, oxidant–antioxidant imbalance and angiogenesis disorders, may play a significant role in the development and progression of bladder cancer. Thus, in this article, we aim to elucidate, using the available literature, the role of inflammatory mediators, prooxidant and antioxidant enzymes and pro-angiogenic and anti-angiogenic factors in the pathogenesis of BC.

## 2. Oxidative Stress and Bladder Cancer

Oxidative stress plays a crucial role in the development mechanism of many diseases, including bladder cancer. The compounds associated with oxidative stress and bladder cancer development are presented in Table 2.

Previous studies confirmed that a decrease in the production of reactive oxygen species (ROS) can prolong life span and prevent tumour development, while ROS overproduction promotes carcinogenesis and tumour progression [75,76,77,78]. ROS may damage proteins, nucleic acids and lipids. It has been shown that the course of cancer may be associated with the overproduction of the lipid peroxidation products [19,79]. Similarly, Lepara et al. (2020) found that patients with BC were characterised by increased serum levels of malondialdehyde (MDA) compared to the control group [80]. MDA is a product membrane phospholipids peroxidation and generated by increased arachidonic acid metabolism due to elevated levels of cyclooxygenase-2 (COX-2). BC cells were characterised by existing *COX-2* expression, while in normal urothelial cells, the *COX-2* expression was undetectable [58]. Therefore, *COX-2* may be involved in tumorigenesis in the bladder. A previous study showed that an advancing grade and T stage of superficial transitional cell carcinoma of the bladder were associated with a high level of *COX-2* expression [59]. Moreover, the level of *COX-2* expression was inversely correlated with the existing recurrence of non-muscle invasive bladder cancer (NMIBC) [60]. The peroxidation of membrane phospholipids and MDA generation may alter membrane permeability and microcirculation. As a consequence of membrane dysfunction, further adhesion of granulocytes to the endothelium was observed. The adhesion may activate the xanthine–oxidase and cause enhanced hydrogen peroxide production in a vicious circle. Moreover, ROS may also activate NF-κB (nuclear factor kappa-light-chain-enhancer of activated B cells), which causes the production of proinflammatory cytokines, which in turn enhance inflammation and, therefore, the overproduction of further ROS [81]. Another biomarker reflecting the level of oxidative stress is 8-iso-prostaglandin F2 α (8-iso-PGF2 α), which is formed by free radical-mediated oxidation of arachidonic acid. Szymańska et al. (2020) found that the median of 8-iso-PGF2 α in urine was 1.5-fold higher in patients with BC than controls. However, no correlation was observed between 8-iso-PGF2 α level and the degree of malignancy and invasiveness of BC [82].

ROS may be produced by NADPH oxidase (NOX) activity. Thus, abnormalities of NOX function were observed in the course of BC. The overexpression of *NOX-4* was found in patients with high-grade, superficially and deeply invasive carcinomas, while in low-grade and non-invasive phenotypes, it was not detected [61]. Interestingly, in vitro studies confirmed that *NOX-4* silencing was associated with the decreased production of ROS and led to the suppression of cancer cell growth by p16-dependent cell cycle arrest at the G1 phase. Thus, disorders of NOX-4 may play a crucial role in the molecular mechanisms involved in urothelial carcinogenesis’s early steps [61]. 

The subsequent interesting enzyme associated with oxidative stress is inducible nitric oxide synthase (iNOS), which generates nitric oxide (NO) under stimuli-dependent conditions. Previous studies confirmed the dualistic character of the NO, where low concentrations (pico- to the nanomolar range) lead to tumour promotion while higher concentrations induce apoptosis leading to tumour suppression (micromolar range) [61,62,63,64]. Patients with BC were characterised by a higher level of NO in bladder cancer tissue, urine and serum [64,65]. Interestingly, the NO level was decreased in patients after surgical treatment to the level observed in controls [64]. The high level of NO correlated with increased expression of *iNOS* detected in bladder tumoral tissue [62]. Moreover, Sandes et al. (2012) found that overexpression of *iNOS* also correlates with the transition to more advanced stages of bladder cancer [62].

The development of oxidative stress in the course of BC is also associated with disturbances in antioxidant enzymes, including superoxide dismutase (SOD), catalase (CAT), glutathione peroxidase (GPx and paraoxonases (PONs). SOD catalyses the dismutation of the superoxide radical into ordinary molecular oxygen and hydrogen peroxide. Patients with malignant tumours were characterised by reduced SOD activity compared to benign tumours and lower *SOD* expression in invasive transitional cell carcinomas than in superficial transitional cell carcinoma [83,84,85]. Similarly, Jeon et al. (2007) confirmed that cancer tissue was recognised by *SOD* expression about 30% lower than normal bladder tissue [86]. Moreover, Wieczorek et al. (2017) showed that the *SOD2* expression in peripheral blood leucocytes and SOD1 activity in erythrocytes were decreased in patients with BC at diagnosis [69]. Moreover, the Ala9Val polymorphism of *SOD2* may modulate the risk of BC development. Ala at position 9 ensures a typical amphilical helical structure, which is essential for its effective transport into mitochondria, while Val at the position disrupts this structure. The Val/Val genotype of the SNP was associated with nearly double the risk of BC. Interestingly, the coexistence of Val/Val genotype of *SOD2* SNP and occupational exposure to polycyclic aromatic hydrocarbons thrice increased the risk while the coexistence of a Val/Val genotype and smoking cigarette increased the BC risk sevenfold [70]. The level of SOD in serum and whole blood was decreased in patients with BC comparing to the control group [66,87]. Moreover, SOD level in serum and blood was additional negative correlated with the stage of bladder cancer—the lowest level of SOD was observed in patients with the most advanced cancer [87]. Likewise, serum and blood level of CAT was lower in patients with BC than controls [66]. CAT catalyses the decomposition of hydrogen peroxide into water and oxygen. The cancerous BC tissue showed a decreased expression and activity of CAT as compared to normal bladder tissue [83,85]. Similarly, Jeon et al. (2007) confirmed that the bladder cancer tissue showed a *CAT* expression about 30% lower than normal bladder tissue [86]. On the other hand, CAT activity in serum patients with BC was increased compared to controls [67]. Moreover, *CAT* expression in leucocytes was higher in patients with recurrence after 1 year from transurethral resection than in these patients at diagnosis moment. Thus, the low expression of *CAT* may contribute to BC recurrence in the early prognosis [69]. Interestingly, the in vitro study showed that the highly metastatic (253J BV cell line) bladder tumour cells were characterised by significantly lower CAT activity than nonmetastatic (253J cell line) bladder tumour cells. In addition, the decrease of CAT activity was accompanied by an increase in *SOD2* expression, resulting in an increase in hydrogen peroxide level in 253J BV cells. Moreover, the metastatic cells showed an elevated expression of the pro-metastatic and proangiogenic factors: matrix metalloproteinase 9 (*MMP-9*) and vascular endothelial-derived growth factor (*VEGF*). Thus, metastatic bladder tumour cells presented an altered antioxidant expression profile, which led to ROS overproduction. The high level of ROS generation induced redox-sensitive pro-tumorigenic and pro-metastatic genes such as *VEGF* and *MMP-9,* whereas the cell model characterising *CAT* overexpression showed a decrease in MMP-9 activity and suppressed the clonogenical activity, leading to the inhibition of the metastatic effect of cells [68]. Schäfer et al. (2007) found that the GC-rich *VEGF-A* promoter region spanning −88 to −50 is indispensable for basal and oxidative stress-triggered this promoter activity. Oxidative stress increases DNA–protein complex formation at the −88/−50 site in gastric cancer cells. The results suggest that enhanced binding of Sp1/Sp3 to the *VEGF-A* promoter may be a crucial mechanism of transactivating the *VEGF-A* expression induced by oxidative stress [88]. Sp1 is a transcriptional factor and allowed regulation of expressed “housekeeping genes” and genes associated with growth and differentiation. Sp3 activates GC-rich DNA elements. However, it may also act as a transcriptional repressor of other transcription factors binding to the same element [89,90]. As a result of oxidative stress, gastric cancer cells were characterised by enhanced binding Sp1 and Sp3 to two GC-boxes at −73/−66 and −58/−52. The increased binding of Sp1 might be the result of Ras-dependent activation of the Raf → MEK1 → ERK1/2 kinase. Finally, ERK1/2 may lead to phosphorylation of Sp1 and thus may cause an increased binding of Sp1 with the *VEGF* promoter region and its expression upregulation [91]. Interestingly, Schäfer et al. (2007) observed that oxidative stress leads to enhanced Sp1 transactivating capacity but not Sp3. Thus, the results suggest differences in the regulatory mechanisms of the transcription factors in response to oxidative stress [88]. In the cause of MMP, a previous study showed that oxidised low-density lipoprotein (oxo-LDL) induces *MMP-9* expression [92]. Molecular analysis showed that the *MMP-9* 5′-proximal promoter region consists of putative binding sites for AP-1 (−79 and −533), NF-κB (−600), Sp1 (−558) and PEA3 (−540) [93]. Therefore, the NF-κB and AP-1, activation is crucial in *MMP-9* expression, leading to remodelling of the extracellular matrix and membrane degradation [94]. AP-1 consists of c-Jun and c-Fos protein families [93,95,96]. The obtained results demonstrated that ROS, including oxLDL, induced *proMMP-9* expression via MEK1/ 2-p42/p44 MAPK, PI3K/Akt and JNK1/2 (c-Jun N-terminal kinase) pathways, linked to activation of transcription factor AP-1 (c-Fos and c-Jun) [92,97]. 

The next important antioxidant enzyme is GPx, which reduces lipid hydroperoxides to their corresponding alcohols, and free hydrogen peroxide to water. Lower GPx activity was observed in erythrocytes and in bladder cancer tissues compared with the bladder tissues of patients without tumour [84,98,99]. On the other hand, Wieczorek et al. (2017) reported that the activity of GPx1 in erythrocytes and *Gpx1* expression in leucocytes was higher in patients at diagnosis of BC compared to the control group [69]. In the case of patients with recurrence after 1 year from transurethral resection, the activity of GPx1 in erythrocytes and GPx3 in plasma was lower than these patients at the moment of diagnosis. Moreover, Ichimura et al. (2004) found that the SNP of *GPx1*, which caused the substitutes Pro to Leu at codon 198 (Pro198Leu), may modulate BC risk [100]. The frequency of the Pro/Leu genotype of the *GPx1* gene was higher in patients with BC than in the control group, and additionally, the Pro/Leu genotype was associated with the advanced tumour stage (significantly more common in cases of T2-T4 tumours than T1 tumours). Thus, patients with the Pro/Leu genotype were characterised by an increased risk of advanced disease (T2-T4) compared with patients with the Pro/Pro genotype [100]. The next study confirmed that the Pro198Leu polymorphism may also be associated with bladder cancer recurrence risk. Zhao et al. (2005) found that the patients with superficial bladder cancer with Pro/Pro genotype of *GPx1* were characterised by longer overall recurrence-free survival but only in individuals younger than 64 years and in women [101]. 

The subsequent enzymes involved in antioxidative defence are paraoxonases (PON), including PON1, PON2 and PON3. PON1 and PON3 are localised in the plasma, whereas PON2 is in the plasma membrane, endoplasmic reticulum, nuclear envelop and inner mitochondrial membrane [74,102,103,104]. The patients with diagnosed BC characterised the decreased activity of PON1 in serum compared to the control group [71]. Moreover, these patients showed a low concentration of PON1 in serum as compared to controls, which were associated with a higher level of the chemokine (CC motif) ligand 2 C-reactive protein. Interestingly, the lower PON1 concentration was observed in patients with tumour recurrence compared to patients without tumour recurrence [72]. The next study showed that the polymorphism localised in the *PON1* gene, which results in Q/R substitution at codon 192, may modulate BC risk. The Q/Q genotype frequency was lower in the patient group than controls, while the R/R genotype was more common in BC patients. Moreover, the R/R genotype was also associated with invasive growth pattern, perineural invasion, distant metastasis [73]. In the case of *PON2*, the study confirmed that patients were characterised by an increased expression in cancer tissue compared to normal bladder tissue [74]. Moreover, *PON2* expression in BC patients’ urinary exfoliated cells was higher than in patients affected with tumours invading subepithelial connective tissue or extending outside the bladder (T1-T3). Since PON2 is located in the mitochondria, the enzyme protects cells against ROS overproduction within the mitochondrial respiratory chain. Thereby, the release of cytochrome c and caspase activation is minimised, which finally inhibits apoptosis induction [103,105,106,107]. The increased expression of *PON2* in bladder cancer may contribute to apoptotic escape of tumour cells [103,108,109,110]. Similarly, in vitro studies confirmed that the human urinary bladder cancer cell line T24 with induced *PON2* overexpression showed an increased cell proliferation. Thus, PON2 activity may play a crucial role in promoting bladder tumorigenesis [74]. 

To sum up, these disorders involved in oxidative stress could be used to develop new biomarkers of bladder cancer (Table 3).

The ROS overproduction described above may lead to cancer transformation of cells. ROS participates in signal transduction and is involved in the regulation of cell proliferation, apoptosis and tumorigenesis by modulating the expression of transcription factors, enzymes and structural proteins [111,112,113,114]. Increased ROS level can lead to DNA oxidative damage, including DNA strand breakage, DNA–DNA crosslink or DNA–protein crosslink. The gene damage associated with cancer development includes *Ras* and *p53* genes. Previous studies confirmed that *Ras* mutation was identified in about 30% of BC patients, whereas the *p53* mutation is associated with over 50% of BC patients [115,116]. Moreover, ROS may modulate the methylation status of the promoter region of genes associated with carcinogenesis. ROS expositions can cause hypermethylation of tumour suppressors and hypomethylation of oncogenes, which are involved in cancer initiation [117,118]. Moreover, ROS might stimulate ROS/MAPK and ROS/Keap1-Nrf2-ARE signalling pathways associated with promoting or suppress BC cell proliferation, migration and invasion [119,120,121]. However, keep in mind that ROS is a double-edged sword. The moderate level of ROS can promote cancer cell survival, whereas excessive levels kill them. High ROS level may lead to lethal oxidative damage to DNA and trigger cancer cell death. On the other hand, a moderate ROS level may initiate the process involved in cancer transformations [122].

## 3. Inflammation and Bladder Cancer

Chronic inflammation may lead to carcinogenesis. The inflammation microenvironment is characterised by a high count of macrophages and lymphocytes. As a result of macrophage and lymphocytes activities, large amounts of ROS and RNS, including peroxynitrite, are generated. Peroxynitrate is a mutagenic compound reacting with DNA and may lead to DNA mutations of proliferating cells, including bladder epithelial cells [123,124]. Moreover, macrophages and lymphocytes may release tumour necrosis factor-alpha (TNF-α) and macrophage migration inhibitory factor, following exacerbated DNA damage [125]. Migration inhibitory factor impairs p53-dependent protective responses and may cause the accumulation of oncogenic mutations [126]. The second pathway associated with migration inhibitory factor and contributing to tumorigenesis is the Rb-E2F pathway. In normal condition, the RB protein binds to and inhibits E2F. The complex RB-E2F prevents cells with damaged DNA from going through the G1 phase and entering the S phase, thanks to which damaged genes are not replicated. The high migration inhibitory factor level associated with chronic inflammation prevents Rb’s inhibition of E2F and leads to elevated proliferation and tumorigenesis [127].

The factor that induced inflammation may be oxidative stress and may stimulate the production of proinflammatory cytokines, including interleukins (ILs) and TNF-α, which are involved in cancer development. The characteristics of selected cytokines involved in the development of bladder cancer were presented in Table 4.

**Figure 1:**
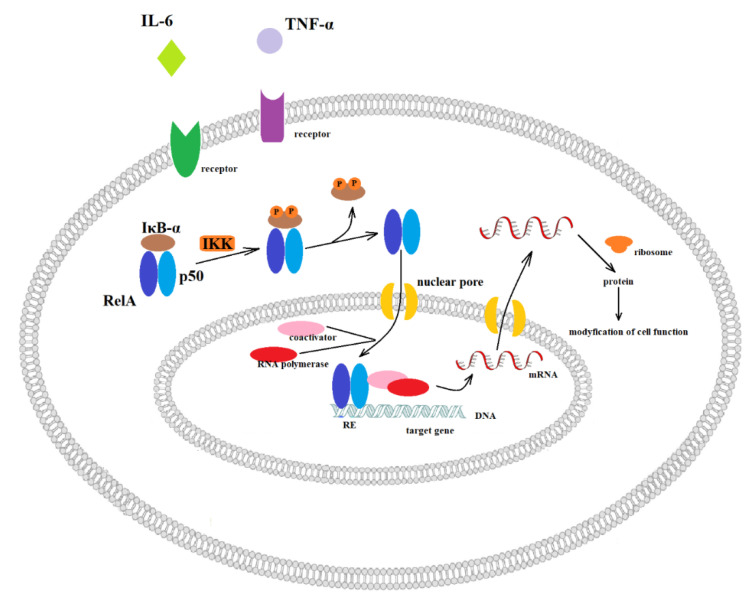
Canonical activation of NF-κB. Inactive NF-κB heterodimer consisting of RelA and p50 proteins are located in the cytosol where it is complexed with the inhibitory protein IκBα. Through the integral membrane receptors, the various extracellular signals can activate the enzyme IκB kinase (IKK, inhibitor of nuclear factor-κB). Then, IKK phosphorylates the IκBα protein, which leads to ubiquitination and dissociation of IκBα from NF-κB. In the next step, the activated NF-κB is translocated into the nucleus, where it binds to specific sequences of DNA called response elements (RE). Then, coactivators and RNA polymerase are recruited, and finally, DNA is transcribed into mRNA. In turn, mRNA is translated into protein, resulting in a change of cell function.

Cytokines, including IL-6, IL-11, IL-27, interferons (IFN-α/β/γ) and their receptors, may mediate the malignant transformation of urothelial cells and the progression of BC via activation of JAK-STAT3 (Janus kinase/signal transducer and activator of transcription 3) pathway [201,202]. JAKs are signalling proteins involved in transducing signals due to tyrosine phosphorylation of STAT-3 [203]. The activation of the JAK-STAT3 pathway may be responsible for the growth and survival of bladder cancer cells [155,156], while the STAT3 silencing may cause the suppression of T24 bladder cancer cell proliferation [204]. The previous study also confirmed that STAT3 may might be involved in the invasion, migration and BC progression [205,206]. Moreover, activated STATs by cytoplasmic p27 induces a TWIST1 (twist-related protein 1)-dependent epithelial–mesenchymal transition (EMT), causing an increase in the invasion and metastasis of BC [207]. The next pathway associated with inflammation and bladder cancer is the activation of NF-κB. In the canonical pathway, NF-κB may be activated via proinflammatory cytokines, including TNF-α or IL-6. The canonical pathway is presented in Figure 1.

Levidou et al., (2008) found that nuclear *NF-κB* expression is associated with histologic grade and T category in bladder urothelial carcinoma [191]. Thus, the reduction of NF-κB activation causes an increase in bladder cancer cells’ sensitivity towards chemotherapeutic treatment [192,193]. In addition, NF-κB activation may also mediate angiogenesis and metastasis of bladder transitional cell carcinoma through the regulation of proinflammatory cytokine, IL-8 [169]. Under basal conditions, NF-κB is associated with IκB, which prevents NF-κB translocation to the nucleus. Activation of NF-κB occurs as a result of IκB-α phosphorylation at 32Ser and 36Ser position and then its polyubiquitination, and finally degradation by the 26S proteasome. Consequently, liberated NF-κB is transported to the nucleus and impacts transcription of the *IL-8* gene [170]. Interestingly, the level of *IL-8* expression may be associated with the metastatic potential of human transitional cell carcinoma [171]. Moreover, the bladder transitional cell carcinoma tumours are heterogeneity, and thus part of the cancer cells are exposed hypoxia, which in turn increases anaerobic metabolism resulting in enhanced production of acidic metabolites [172,173,174,175,176,177,178]. The hypoxia and subsequent acidosis can promote malignant progression by increasing the expression of *IL-8*, which results in phosphorylation of IκB-α. Hypoxia and acidosis are stimuli that activate this phosphorylation [179,180,181]. Therefore, the inhibition of NF-κB by mutated IκB-α with substitutions at the serine 32 and serine 36 residues led to the reduced expression of *IL-8,* in resulting in a decreased angiogenesis, invasion, and metastasis in human ovarian and prostate cancer models [182,183]. The -251 T/A polymorphism in the promoter region of *IL-8* may modulate its expression and risk BC development. The A/A genotype of the SNP was associated with twice-increased BC risk; moreover, the same genotype reduced the recurrence risk after immunotherapy [184]. On the other hand, Wu et al. (2018) found that the T/T genotype of the −251 T/A polymorphism increased the BC risk, particularly in people who ever smoked [208].

As noted above, NF-κB might be activated by TNF-α, which shows dualistic nature—pro- and anti-tumorigenic factor. Thus, its role in cancer progression is still a matter of debate. TNF-α at a high concentration acts as an anti-tumorigenic factor [128], while the moderate concentration of TNF-α may stimulate angiogenesis, metastasis and cause damage to the DNA [129]. In vivo studies confirmed that TNF-α showed cytotoxic effects and induced apoptosis [130]. On the other hand, the pro-tumorigenic effect of TNF-α may be involved in inhibiting DNA repair mechanisms [131]. TNF-α exerts its dualistic character through activating distinct signalling pathways, including NF-κB, JNK and apoptosis. NF-κB is a main cell survival signal, which is anti-apoptotic, while the JNK pathway and caspases activation lead to cell death. TNF-induced NF-κB activation causes the transcription of A20, cIAP-1, cIAP-2, Bcl-xL, XIAP and IEX-1L, which show anti-apoptotic properties. On the other hand, TNF-α may induce apoptosis by binding TNFR-1. As a consequence of TNFR-1 activation, the TRADD, RIP, FADD and caspase-8 complex forms. Then caspase-8 activates the effector caspases, including caspases-3 and -7 and the endonucleases, destroying cell component proteins, causing fragmentation of DNA and, eventually, apoptotic cell death. TNF-α may also induce apoptosis through the mitochondria-mediated (intrinsic) apoptosis pathway. It is made possible by caspase-8 activating the BCL-2 interacting domain (Bid). The cleavage of Bid causes a loss of mitochondrial membrane potential and release of cytochrome c. Consequently, a sequence of events leading to cell death is induced [128]. Moreover, in vitro study confirmed that anchorage-dependent nontumorigenic rat urothelial cells (MYP3) after pretreatment with hydrogen peroxide and then exposition to TNF-α for 1 week showed an increased number of colonies as compared to untreated control cells. Moreover, the treatment with TNF- α alone caused colony formation and led to an above 8-fold increase in the intracellular level of hydrogen peroxide. A vicious circle phenomenon appears; oxidative stress induces TNF-α and then activated TNF-α generates a greater amount of ROS. In turn, the antioxidant (alpha-tocopherol) application caused a reduction of the colony number induced by TNF-α [132]. TNF-α belongs to a group of proinflammatory cytokines and is involved in cancer growth and progression [133]. TNF-α is involved in angiogenesis by the regulation of thymidine phosphorylase [134], whereas the enzyme is associated with bladder cancer progression [135]. Patients with BC were characterised by an increased protein level of TNF-α in urine [136]. Moreover, BC patients with or without schistosomiasis were characterised by a high serum level of TNF-α. Interestingly, a higher TNF-α concentration was detected in T3 and T4 advanced-stage patients compared to T1 and T2 early-stage patients, indicating that TNF-α level may contribute to the BC progression [137]. The meta-analysis showed that the −308 A/A+G/A genotype of *TNF-α* also correlated with the bladder cancer grade [138]. The SNP is localised in the promoter region of *TNF-α* and may impact its expression level. A previous study suggested that the allele A of −308 G/A polymorphism was associated with an increased expression level of *TNF-α* [139]. The +488A and −859 polymorphisms of *TNF-*α were associated with BC risk. Moreover, the occurrence of investigated polymorphisms was correlated with the BC tumour grade [140]. −308 G/A polymorphism was associated with the tumour stage and grade of bladder cancer in the Korean population. Therefore, the study suggests that the tested polymorphism may involve angiogenesis regulation, which is essential for the invasion and metastasis of cancer [141,142]. Additionally, smokers or people who had ever smoked and carried the A/A genotype of *TNF-α* −308 and the T/T genotype of *IL-8* −251 polymorphism were characterised by a higher risk of BC than non-smokers [131]. Previous results showed that people who had ever smoked had higher circulating TNF-*α* level than people who had never smoked [143]. The animal study also confirmed that cigarette smoking causes activation of systemic inflammation resulting in upregulated expression of *TNF-α* [144]. Thus, smoking patients are at increased risk of developing BC. Moreover, −863C>A polymorphism of *TNF-α* also modulated the risk of BC development. Furthermore, the C/A+A/A genotype variant of the studied polymorphism was more common in patients with grade I (44.6%) than grade III (29.8%). The subsequent study showed that the C/C genotype of the −1031T/C (rs1799964) polymorphism of *TNF-α* was associated with an increased risk of BC development [145]. Interestingly, TNF-α may interact with MMP-9, which involved in an invasion and migration of cancer cells. Previous studies showed that MMP-2 and MMP9 are associated with a high stage and grade of bladder cancer [146,147]. Moreover, Nutt et al. (2003) found that MMP-9 activity was increased 17-fold in patients with invasive tumours compared to controls, indicating the crucial role of this enzyme in bladder cancer [147]. The cellular production of MMP-9 was induced by proinflammatory cytokines, particularly TNF-α [148]. *In vitro* study confirmed that urinary bladder cancer HT1376 cells were, via increased secretion of MMP-9, stimulated by TNF-α [149,150]. TNF-α also impacted the transcription of MMP-9 by stimulating the 5′-flanking promoter activity of MMP-9. The stimulated 5′-flanking promoter included three potential TNF-α-binding sites, NF-κB, AP-1 and Sp-1. Lee et al. (2008) confirmed that these binding sites are essential for TNF-α mediated activation of *MMP-9* gene transcription in HT1376 cells [150]. Moreover, the study showed that ERK1/2 (extracellular signal-regulated kinase 1/2) and MAPK (p38 MAP kinase) may be involved in the regulation of TNF-α-induced *MMP-9* expression in HT1376 cells [150].

**Figure 2:**
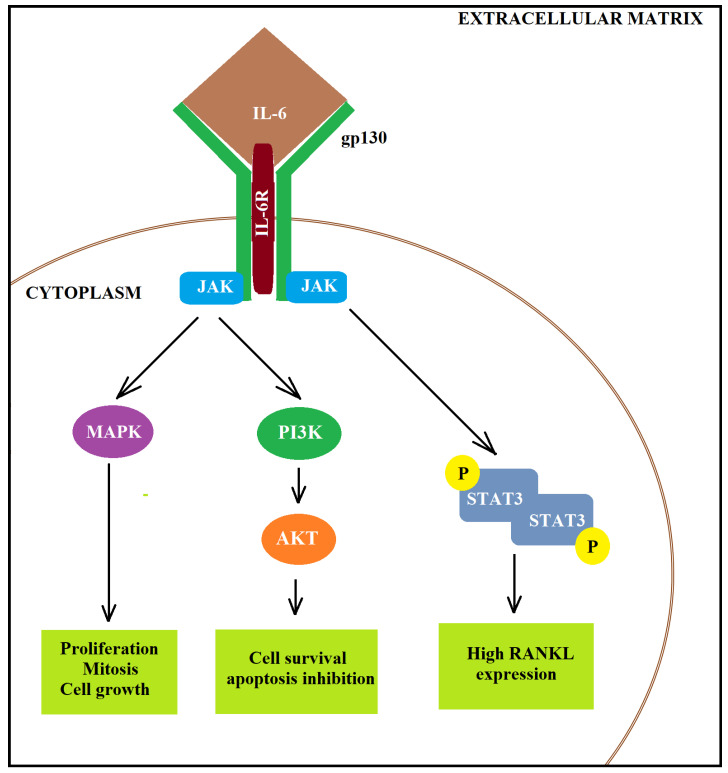
Activation of IL-6 signalling pathway. IL-6 may activate AKT via increased JAK-dependent PI3K activity, causing increased cell survival and anti-apoptosis properties. To this end, firstly, IL-6 binds to the IL6R. Then, the complex binds to the membrane-bound gp130 dimer to form an IL-6 trans-signalling complex and activates (i) the JAK-dependent MAPK, (ii) the JAK-dependent PI3K or (iii) the JAK-dependent STAT3.

In addition to the interleukin 8, IL-6 is also involved in the development mechanism of BC. The bladder cancer specimens showed the *IL-6* overexpression at both mRNA and protein levels compared to non-malignant tissues [154]. Moreover, a high serum level of IL-6 was associated with metastasis and poor prognosis of bladder cancers [155]. The level of IL-6 in urine was also increased in patients with locally advanced bladder transitional cell carcinoma compared to patients with NMIBC [154]. Moreover, IL-6 may induce proliferation and prolong cancer cells’ survival [156]. In turn, blocking IL-6 resulted in a reduction of BC tumour growth and invasive ability due to a decrease in cell proliferation, lower epithelial–mesenchymal transition, reduced DNA methyltransferase 1 expression and attenuated angiogenesis [154]. IL-6 may activate protein kinase B (AKT) via increased JAK-dependent PI3K (phosphoinositide 3-kinase) activity causing an increase in cell survival and anti-apoptosis properties. To this end, firstly, IL-6 binds to the IL6R. Then, the complex binds to the membrane-bound gp130 dimer to form an IL-6 trans-signalling complex and activates (i) the JAK-dependent mitogen-activated protein kinase (MAPK), (ii) the JAK-dependent PI3K or (iii) the JAK-dependent STAT3 [157]. IL-6 is involved in the activation of the JAK/STAT3 (Janus kinase/signal transducers and activators of transcription 3) pathway [158,159] and activated STAT3 signalling may contribute to oncogenesis by promoting proliferation and EMT [160,161]. In addition, activated STAT3 signalling may predispose urothelial basal cells toward the progression into invasive bladder cancer [99]. Chen et al. (2008) showed that IL-6 inhibition decreased STAT3 activity and, as a result, led to increased *E-cadherin* and decreased *VEGF* and *MMP-9* expressions [202]. Therefore, IL-6 by activation of STAT-3 signalling may stimulate the invasiveness of BC. The IL-6 signalling pathway is presented in Figure 2.

On the other hand, IL-6 also presenst a fairer face that is associated with anti-tumour adaptive immunity [162,163]. IL-6 is involved in boosting T cell trafficking to lymph nodes and to tumour sites, where they have the opportunity to become activated and execute their cytotoxic effector functions, respectively. Dendritic cells in the lymph node may produce a large amount of IL-6 that impact the activation, expansion, survival and polarisation of T cells during an immune response [164]. Moreover, IL-6 may be contributing to promoting T cell proliferation following T cell receptor stimulation [165,166]. IL-6 further protects T cells from apoptotic death by the induction of BCL-2, BCL-xL cFos and JunB [165,167]. Moreover, IL-6 may also resculpt the T cell immune response, shifting it from a suppressive to a responsive state that can effectively act against tumours [168].

The subsequent interleukin associated with bladder cancer is IL-17. Similar to IL-6, IL-17 may induce tumour growth through an IL-6-Stat3 signalling pathway [185]. Moreover, the C/C genotype of -174 G>C polymorphism localised in the *IL-6* gene was associated with the increased risk of BC in ever smokers and not in never smokers with the highest BC risk occurring current smokers [209,210]. The higher risk of BC in smokers than not in never smokers is associated with increased production of proinflammatory cytokines, including TNF-α, IL-8 and decreased production of anti-inflammatory cytokines, including IL-10. Moreover, gender analysis showed that the C/C genotype of the −174G>C polymorphism was associated with the risk of BC occurrence in the men group but not in women [210]. Similarly, the metanalysis confirmed that the occurrence of a dominant model (C/C+G/C vs. G/G) and recessive model (C/C vs. G/C+G/G) was associated with high BC risk in the Asian population [211]. Ebadi et al. (2014) also confirmed that the C/C and G/C genotype may be associated with an increased BC risk [212]. Interestingly, the G/C and C/C genotypes of the SNP were correlated with the near twice-increased progression risk in patients with non-muscle-invasive BC [209]. Therefore, the −174G>C polymorphism of the *IL-6* may play a crucial role in bladder cancer development. The change from G to C at position 174 of *IL-6* gene leads to creating a potential binding site for the transcription factor NF-1. This transcription factor may act as a repressor of gene expression, causing reduced production of IL-6 [139,213]. The wild genotype of the *IL-6* gene ensures high production of the interleukin causing the activation of IL-4 and inhibition of IL-12 mediated Th1 type cell differentiation [151,214]. Thus, the C/C genotype of the SNP may favour an increase of the inflammation and, consequently, development of the BC. On the other hand, the G/C+C/C variant of the *IL-6* improved a 5-year overall and disease-specific survival in patients with invasive BC [209]. Moreover, in the Indian population, the G/C+C/C variant genotypes were more common in patients with grade I than grade III tumours. Thus, the clinical study suggests that −174G>C substitution contributed to protection against the risk of urinary bladder cancer in Indian [164]. 

On the other hand, the production of IL-17 may be induced by IL-23. The previous study suggests that IL-23 induces differentiation of naive CD4+ T cells into highly pathogenic helper T cells (Th17/ThIL-17), which produce IL-17, IL-17F, IL-6 and TNF-α [215,216]. The activated IL-17 stimulates fibroblasts, endothelial cells, macrophages and epithelial cells to produce proinflammatory cytokines, including IL-1, IL-6, TNF-α, NOS-2, metalloproteases and chemokines, leading to the induction of inflammation [186]. Therefore, the inhibition of IL-23/IL-17 axis may be a potential aim of anticancer therapy. The decreased levels of IL-17 and TGF-β in peripheral blood may be used as indicators for the worse prognosis of patients with BC [187]. The worse prognosis may be correlated with the high expression of *IL-17,* which may play a crucial role in angiogenesis promotion [188]. IL-17 may increase MMP-9 production to promote angiogenesis and tumour growth [189]. Moreover, both tumour tissue and serum of patients with urothelial bladder cancer were characterised by an increased expression level of *IL-17* and *IL-23R* as compared to the tissue and serum of controls. Moreover, the protein levels of IL-17 and IL-23R were correlated with the clinical stage and lymphatic metastasis occurrence [190]. Interestingly, an in vitro study showed that MB49 cells with knockout *IL-17* (−/−) were characterised by reduced growth [185]. In addition, the TT genotype and T allele of rs763780 and A/A genotype and A allele of rs2275913 polymorphism localised in *IL-17* gene were associated with an increased risk of BC occurrence in a Chinese Han population. Similarly, Lima et al. (2015) confirmed that the A/A genotype of -197G/A (rs2275913) polymorphism was associated with an increased BC risk [145]. However, only rs763780 polymorphism was associated with invasion of bladder cancer [217]. On the other hand, in those produced by Th17 cells, IL-17 was characterised by not only an oncogenic potential in tumorigenesis by regulating tumour angiogenesis and enhancing tumour immune evasion but also exerts anti-tumour functions [218]. The anti-tumorigenic properties include enhancing natural killer (NK) cells and cytotoxic T lymphocytes (CTLs) activation and the recruitment of neutrophils, NK cells and CD4+ and CD8+ T cells to tumour tissue [218,219]. Moreover, previous studies confirmed that IL-17 might play a role in inhibiting tumour cell invasion. IL-17 might recruit and promote infiltration of neutrophil N1, which plays a crucial role in anti-tumour immunity [220,221,222]. IL-17-induced neutrophils might be involved in the destruction of tumour cells [223]. The anti-tumorigenic action of IL-17 produced by neutrophils N1 was associated with increasing T cell production and activation and amplifying T cell proliferation [224,225,226].

The TGF-β mentioned above is a proliferation inhibitor in epithelial cells and acts as a tumour suppressor [194]. Moreover, continuous expression of *TGF-β* is involved in cancer progression [195]. The T/T genotype of +28C>T polymorphism of *TGF-β* was associated with a reduced risk of recurrence after immunotherapy [196]. Interestingly, similar to TNF-α, TGF-β is also characterised by a dual nature. TGF-β is a multifunctional factor and is involved in the regulation of apoptotic, angiogenic, immunogenic and anti-tumorigenic responses [197,198]. TGF-β suppresses NK cell growth and activity as well as inducing the synthesis of IFN-γ [199]. On the other hand, in the case of pancreatic ductal adenocarcinoma, results confirmed that TGF-β promotes angiogenesis and immune suppression by rendering chemoresistance and facilitating invasion and metastasis [200]. The subsequent cytokines associated with bladder cancer are IL-5, IL-20 and IL-28A. Recently, the increased expression level of *IL-5*, *IL-20* and *IL-28A* genes have been shown in patients with muscle-invasive bladder cancer. Moreover, the studies confirmed that cytokines might increase *MMP-2* and *MMP-9* expression and activation of NF-κB and AP-1, which regulate the *MMP-9* promoter in muscle-invasive and non-muscle-invasive bladder cancer. In vitro studies also showed that IL-5, IL-20 and IL-28A may lead to activation of MAPK and JAK-STAT pathways [153]. 

In addition to pro-tumorigenic cytokines, anti-tumorigenic cytokines are distinguished. IL-12 is a pro-inflammatory cytokine characterised by anti-tumorigenic properties produced by dendritic cells and macrophages. It plays an essential role in the recruitment and functions of cytotoxic effector cells of the immune system, including CD8+ T and NK cells [227]. IL-12 is involved in the activation of CD8+ T and NK cells. Activated Th1 and NK cells proliferate and infiltrate into the tumour, where Th1 cells support the effector functions of tumour-specific cytotoxic T cells. Moreover, cytotoxic NK and CD8+ T cells secrete IFN-γ, granzyme and perforin, which can induce the apoptosis of cancer cells and control tumour growth [227,228,229]. Therefore, CD8+ T and NK cells recognise and kill cancer transformed cells, and IL-12 is the main contributor to effective anti-tumour immune responses [230]. IL-23 is the next pro-inflammatory cytokine characterised by anti-tumorigenic properties. IL-23 might regulate the function of many immune cells in the tumour microenvironment. IL-23 might increase IL-23R expression on type 3 innate lymphoid cells (ILC3), granulocytes and NK cells, which in turn induce production of the pro-inflammatory cytokines and cytotoxic function [231,232,233]. Additionally, IL-23+ macrophages expanded anti-tumorigenic Th17 cells and promoted tumour-specific immune responses by secreting IFN-γ, CXCL9 and CXCL10 [231,234]. However, Langowski et al. (2006) found that the genetic deletion or blockade of IL-23 in mice caused an increased infiltration of cytotoxic T cells with protective effects against cancer [235]. Moreover, an animal study showed that microbial products might activate intra-tumoral myeloid cells, which in turn produced IL-23 and promoted colorectal neoplasms [236]. Another crucial factor in BC development is the anti-inflammatory cytokine, IL-4, which plays a role in gene suppression, including *TNF-α* and *IL-1*. Moreover, IL-4 is involved in the surveillance and elimination of transformed cells by Th2 development, eliminating pathogens and inhibiting Th1. Thus, the polymorphisms localised in the *IL-4* gene may modulate the risk of BC. A meta-analysis showed that the *IL-4* haplotypes, IL4-589T and IL4-33T, were associated with the patients higher survival rate with the haplotype IL-4-589C-33C. The T/T genotype of *IL-4* polymorphism (rs2243250) was associated with an increased risk of developing multiple BCs [152].

The inflammatory mediators listed in the text can be used in diagnostics (Table 5).

## 4. Modulation of Inflammatory System in Course of BCG Therapy

NMIBC (pTa-pT1) is the most common BC, which constitutes 70–80% of all patients with urothelial carcinoma of the bladder. NMIBC was characterised by a good overall prognosis following transurethral resection in patients with low-risk tumours (pTaG1/2) [237]. On the other hand, this cancer group had heterogeneous tumours characterised by high recurrence (30–80%) and progression (25–50%) rates [238,239]. Unfortunately, the application of complementary therapy with Bacillus Calmette–Guérin (BCG) in patients with T1 NMIBC can also lead to an almost 40% rate of recurrence and 20% of progression at five years [240]. The inflammatory response to BCG involves several steps, including:

(1) Attachment of BCG to the urothelium cells through the interaction between molecules in the bacterial wall and fibronectin in the urothelium;
(2) Internalisation of BCG into resident immune cells, regular cells and urothelial tumour cells through increased macropinocytosis;
(3) BCG-mediated induction of innate immunity, which is characterised by urothelial cells and antigen-presenting cells (APCs) activation and then induction of cytokine and chemokine production (including IL-6, IL-8, granulocyte-macrophage colony-stimulating factor (GM-CSF) and TNF-α that attract granulocytes and mononuclear cells to the bladder. Interestingly, the levels of IL-1β, IL-8, IL-15, IL-18, CXC-chemokine ligand 10 (CXCL10), GM-CSF, CC-chemokine ligand 2 (CCL2) and CCL3 in urine are detectable within the first 24 h after BCG infusion. In addition, in the urinary tract and the bladder, the presence of neutrophils, monocytes, macrophages, T cells, B cells and NK cells was observed after BCG therapy.
(4) BCG-mediated initiation of tumour-specific immunity. APC and urothelial cell activity may lead to BCG antigens presentation of these cells surface via MHC class II. These MHC affect CD4+ T cell receptors, resulting in primarily T helper 1 (TH1) cell immune activation and differentiation. TH1 activation induces the generation of IL-2, IL-12, IFN-γ, TNF-α and TNF-β and leads to the activation of cytotoxic CD8+ T lymphocytes, which destroy cancer cells. On the other hand, in the BCG therapy response, the production of IL-4, IL-5, IL-6 and IL-10 by primary T helper 2 (TH2) was associated with BCG non-responsiveness and cancer progression [241].

Repeated infusions trigger a robust immune response that increases in intensity throughout BCG therapy. BCG therapy, including several series of BCG given alone or in combination with conventional chemotherapy, was characterised by better results of patients with BC compared to transurethral resection of bladder tumour [242,243]. Additionally, BCG given in a three-year maintenance schedule to prevent recurrence in patients with intermediate-/high-risk tumours is more effective than 1-year therapy [243,244,245,246]. Thus, BCG immunotherapy has been identified as the gold-standard treatment for NMIBC at high risk of recurrence or progression [241]. Unfortunately, current research has been observing a worldwide BCG deficiency, and thus potential strain substitutions are wanted. A Dutch study showed that the RIVM strain is superior in terms of recurrence-free survival compared to TICE, and additionally, Connaught prevented recurrence better than TICE [247,248]. One of the recent studies also confirmed that both TICE and RIVM strains were superior to Connaught for prolonging DFS (disease-free survival) [249]. Similarly, Witjes et al. (2016) found that maintenance TICE performs better than Connaught in HGT1 patients [250]. On the other hand, Rentsch et al. (2014) showed that Connaught was characterised by greater 5-year RFS (recurrence-free survival) compared to TICE with only a sole induction course [247]. Therefore, TICE seems to reach its optimum response over time, and with maintenance, shows longer DFS. In the case of repeated transurethral resection of the bladder (re-TUR), TICE improved RFS in patients after re-TUR, whereas RIVM provided longer PFS (progression-free survival) and CSS (cancer-specific survival) in patients who had not received re-TUR [249]. Interestingly, the better performance of TICE and RIVM than Connaught may be associated with better tolerability. Therapy using Connaught was associated with a high occurrence of frequencies of gross haematuria and systemic adverse reactions [251].

Interestingly, the risk of recurrence in patients with high-grade T1 tumours receiving BCG therapy may be modulated by tumour size, age and presence of carcinoma in situ [252], peripheral blood neutrophil-to-lymphocyte ratio (NLR), high-grade T1 on re-transurethral resection of bladder tumour (TURBT) [253,254], obesity [255], as well as lymph-vascular invasion [256]. The high NLR in bladder cancer patients treated with BCG was characterised by decreased relapse-free survival and progression-free survival [253,254]. Thus, the high pre-treatment NLR is a negative prognostic marker in patients with NMIBC after BCG therapy. Unfortunately, these results should be handled with caution because no breakpoint could be used as a diagnostic marker [253,254]. The next potential prognostic biomarker may be IL-2. The increased TH-1-induced production of IL-2 was observed in BCG responders vs. non-responders of BCG therapy. Moreover, the increased risk of recurrence of BCG-treated patients was associated with a higher interleukin-6/10 ratio. However, the subsequent study found that IL-2 urine peak level was observed earlier than IL-10 in responders as compared to non-responders [257]. Moreover, BCG therapy may be associated with basophils that secrete IL-4 and then facilitate Th2 polarisation [258]. Interestingly, the percentage of basophils and IL-4 expression level were higher in tumour-draining lymph nodes than non-tumour draining lymph nodes. Moreover, the basophil count was correlated with IL-4 expression in tumour-draining lymph nodes, supporting the role of basophil as the source of IL-4 [259]. Ferro and colleagues (2020) confirmed that the logarithmic transformation of basophils count was associated with a 30% increment in the hazard of recurrence per unit increase of logarithmic basophils count [257].

Previous studies confirmed that systemic immune-inflammation index (SII), which depends on the peripheral lymphocyte, neutrophil and platelet counts, can use as an indicator of predictions [260]. Interestingly, Zhang et al. (2019) compared the SII, NLR, PLR (platelet-lymphocyte ratio) and CAR (C-reactive protein/albumin ratio) to predict the prognosis in BC patients after radical cystectomy. They suggested that the SII was superior to other analysed parameters and was an independent predictor for survival [261]. Similarly, the comparison of SII, NLR, PLR and CAR to select the optimal prognosis marker in BC patients after BCG immunotherapy confirmed that SII was superior to other analysed parameters [260]. Importantly, the previously obtained results have enabled the development of cut-off thresholds for SII in BC patients. In the case of MIBC (muscle-invasive bladder cancer), in patients who underwent a radical cystectomy for MIBC, the SII cut-off level calculated as 843, whereas for NMIBC the value for SII cut-off was 672. Thus, a high SII level was correlated with low cancer-specific survival (CSS) in MIBC patients after radical cystectomy [260,262]. Moreover, the coexistence of a tumour greater than 30 mm and a high SII was associated with a 3.6-fold increase the cancer progression in MBIC patients. On the other hand, the presence of carcinoma in situ and tumour grade (low grade or high grade) were not correlated with NLR, PLR and SII in patients with MIBC [261,262]. Moreover, in the case of patients with BC after radical cystectomy, the preoperative CAR was also a crucial predictor of survival [263].

Interestingly, BCG therapy may modulate the urine and blood levels of cytokines, which may affect recurrence-free survival in NMIBC patients. Patients with NIMBC were characterised by an elevated IL-8 level in peripheral blood leucocyte, which was also associated with tumour recurrence risk, especially in patients who received BCG therapy. The median recurrence-free survival time for BCG-treated NIMBC patients with high baseline IL-8 levels was shorter than those with low IL-8 levels (7.9 vs. >78.4 months, respectively). Moreover, the high level of IL-8 in urine predicted a shorter time to tumour recurrence in NMIBC patients [264]. The subsequent potential biomarker to predict response to BCG treatment may be an expression of *IL-1β*, *IFN-γ*, *HMOX-1* and *GNLY*. BCG infusion caused fast-increased *IL-1β*, *TNF-α* and *IL-10* expression at the first and sixth week, while the expression of *GNLY* decreased at the sixth week. After treatment, patients who responded to BCG therapy were characterised by less *IL-1β* expression than relapsing patients, whereas lower *IFN-γ*, *HMOX-1* and *GNLY* expression was observed in BCG responders as compared to relapsing patients before treatment [265]. The relapse of NMIBC may be the result of prolonged exposure to inflammatory cytokines, which may stimulate tumour growth through the promotion of proliferation, angiogenesis, DNA damage and ROS overproduction [266,267]. On the other hand, patients whose BCG responded with remission showed an increased IL-2 mRNA expression [268]. Moreover, previous studies showed that loss of heterozygosity (LOH) in the *IFN-α* (chromosome 9p21) was associated with increased BCG failure in patients who underwent TURBT. Genetic analysis indicated that polymorphisms localised in genes associated with inflammation may be also used as a potential biomarker of recurrence risk of NIMBC. The C/C genotype of *IL-6* (-174 G>C) polymorphism was associated with decreased recurrence risk in the BCG-treated group. Moreover, the C/C homozygote increased recurrence-free survival (median recurrence-free survival for G/G genotype (37 months) and C/C genotype (60 months)) [139]. In the case of polymorphism localised in the *TNF-α* gene (rs1799964), the C/C homozygote decreased the recurrence risk after BCG immunotherapy [269]. Ahirwar and colleagues (2009) showed that the homozygote T/T of +28C>T *TGF-β* polymorphism and the A/A genotype of +874T>A *IFN-γ* polymorphism were associated with reduced and increased risk of recurrence after BCG therapy, respectively [197]. In northern India, the A/A genotype of -251 T > A *IL-8* polymorphism was associated with a reduced recurrence risk after BCG immunotherapy. The mean recurrence-free survival for G/G, G/A and A/A genotypes was 24, 39 and 53 months, respectively [184].

## 5. Angiogenesis

**Figure 3:**
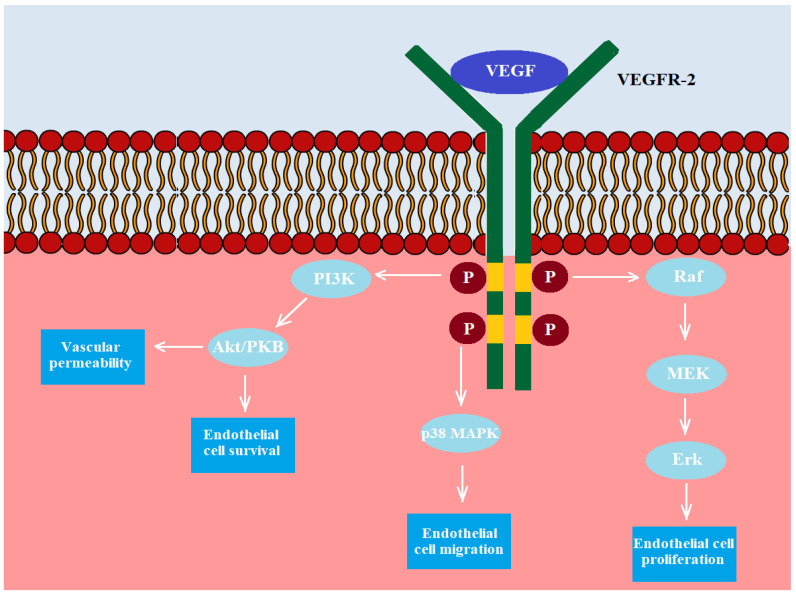
The VEGF receptor (VEGFR) consists of seven domains. VEGF binds to the VEGFR-2 receptor, and then domains within the receptor and below the endothelial membrane are phosphorylated. Next, the phosphorylated domains activate RAF, which then activates MEK1/2, which, in turn, activates ERK1/2. Activated ERK1 or ERK2 either phosphorylates its target cytoplasmic proteins or translocates to the nucleus, where the main targets are transcription factors that regulate proliferation-, differentiation- or survival-related genes. Moreover, the activated VEGFR-2 can activate p38 MAPK and lead to the induction of endothelial cells’ migration. The last pathway associated with VEGFR-R leads to activation of PI3K and then Akt/PKB. Finally, the activation of this pathway leads to increased survival of ECs and vascular permeability.

Angiogenesis is a complex process that is regulated by many factors and leads to the formulation of new capillaries. Thus, the process is crucial for tumour growth and metastasis. In the course of bladder tumour development, an increased production of factors involved in the stimulation of angiogenesis has been observed, including VEGF, basic fibroblast growth factor (bFGF), IL-8 and MMPs [270]. In mammalians, the VEGF family includes five members: VEGF-A, VEGF-B, VEGF-C, VEGF-D and PIGF (placenta growth factor). VEGF-B is involved in embryonic angiogenesis, VEGF-C—in lymphangiogenesis, VEGF-D in the development of lymphatic vasculature surrounding lung bronchioles and PIGF in vasculogenesis. However, the primary role in angiogenesis is played by VEGF-A. VEGF-A is crucial in the increase of the migration of endothelial cells, mitosis of endothelial cells, matrix metalloproteinase activity and αvβ3 activity. Moreover, VEGF-A is involved in the creation of blood vessel lumen and lymphangiogenesis [271]. VEFF-A binds mainly to VEGFR2 expressed on endothelial cells (ECs) and bone marrow-derived endothelial progenitor cells (EPCs). Consequently, the signalling pathways are induced, leading to increased permeability of blood vessels, proliferation and migration of ECs, recruitment of EPCs and maintenance of newly formed vasculature. The mechanism of VEFF-depends on the activation of cell signalling pathways, which is presented in Figure 3.

Previous studies showed that an increased level of *VEGF* expression was observed in the course of BC. Moreover, the VEGF-A level in tissue was correlated with the grade of BC. Thus, abnormal *VEGFs* expression can be used as a prognostic marker in bladder cancer [272]. Donmez et al. (2009) also found that *VEGF* expression was higher in deeper tumours compared to superficial tumours and in invasive tumours compared to non-invasive tumours [273]. In agreement with previous studies, Fauconnet et al. (2009) showed that the *VEGF* transcript level was higher in patients with pT2-T4 than in pTa and pT1 urothelial tumours [274]. Additionally, BC patients with higher mRNA level of *VEGF-A* were characterised by shorter survival without progression than those with a lower level [274]. Similarly, the other study confirmed that mRNA levels of *VEGF* and *VEGFR-1* were higher in bladder cancer than that of normal mucosa. Moreover, the protein expression levels of *VEGF* and *VEGFR-1* were higher in non-muscle-invasive bladder cancer than in muscle-invasive bladder cancer, while the VEGFR-2 protein level was higher in all cancer bladder tissue compared to normal urothelial mucosa [275]. Therefore, the mRNA level of *VEGFR* was correlated to the pathologic stage of BC. On the other hand, Quentin et al. (2004) showed that patients with low-stage superficial transitional cell carcinoma were characterised by a higher level of mRNA expression of *VEGF* than patients with high-stage muscle-invasive carcinomas, and patients with low-grade transitional cell carcinoma had a higher expression level than patient with high-grade tumours [276]. The differences in *VEGF* expression determined in patients with bladder cancer are most likely related to the developmental stage of cancer and methods of collecting tissue samples for expression analysis. Higher *VEGF* mRNA level in superficial low-stage (pTa and pT1) than in advanced muscle-invasive high-stage (pT2 and pT3) transitional cell carcinoma observed by Quentin is compatible with the well-known fact that the stromal stalk of superficial papillary transitional cell carcinoma and the lamina propria underlying preneoplastic flat and papillary urothelial hyperplasia contain a prominent, newly formed microvasculature [276,277]. Moreover, obtained results showed a significant 3-fold decrease of *VEGF* expression in grade 2 transitional cell carcinomas when they had developed a muscle-invasive growth compared to transitional cell carcinoma grade 2 with superficial growth. Thus, this phenomenon of expression change associated with the tumour stage in the group of grade 2 tumours might reflect a crucial event during urothelial carcinogenesis and might indicate conversion of carcinomas at a primarily low level to those with a high malignant potential in the case of transitional cell carcinoma [276]. Similarly, the 3-to 4-fold higher *VEGF* mRNA expression had been confirmed in patients with compared to muscle-invasive transitional cell carcinoma [277,278,279,280]. However, when analysing tissue expression of malignant tumours, false-negative results may be obtained. Fauconnet’s team (2009) paid special attention to the method of collecting material from patients with invasive tumours. pTa and pT1 bladder tumours are usually protruding in the intraluminal region and can be collected using transurethral resection without contamination by surrounding normal mucosa and smooth muscles [274]. The cells of the smooth muscle and the fibroblasts are characterised by lower *VEGF-A* expression than in the epithelial cells [280]. Therefore, a tissue sample of invasive bladder cancer contaminated with the smooth muscle possibly expresses less *VEGF-A*. Aiming to avoid sample contamination by underlying or surrounding the smooth-muscle layer, Fauconnet’s team (2009) obtained a sample of invasive transitional cell carcinoma from surgical specimens of total cystectomy under direct vision [274]. In contrast, the Quentin team used samples immediately after the ran-urethral resection or, rarely, radical cystectomy for analysis of *VEGF* expression. Accordingly, differences in expression levels may be a methodological consequence of collecting tissue material for the experiment [276]. Interestingly, Bernardini et al. (2001) confirmed that the serum level of VEGF-A in urothelial bladder cancer patients was positively correlated with tumour grade, stage, vascular invasion and the presence of carcinoma in situ [281]; moreover, VEGF-A values exceeding 400 pg/mL were highly predictive of metastatic cancer disease. Moreover, the elevated urine level of VEGF-A was associated with recurrence in non-muscle-invasive bladder cancer [282]. The comprehensive analysis of polymorphisms localised in the *VEGF* gene showed that the A/A genotype of −15648A>C (rs833052) polymorphism, T/T genotype of −9228G>T (rs1109324) polymorphism, the T/T genotype of −8339A>T (rs1547651) and the T/T genotype of Ex1-73C>T (5′UTR) (rs25648) polymorphism were associated with increased risk of BC [160]. On the other hand, in the case of IV42 +1378C>T polymorphism localised in the second intron of *VEGF* gene (rs3024994), the heterozygote decreased the risk [283]. An Asian population subgroup study showed that the C/T genotype of rs3025039 SNP and the C/A genotype of rs833052 SNP were more common in patients with BC than homozygote variants. Moreover, in an in silico analysis, *VEGF* expression in bladder carcinoma tissue is higher than in the standard counterpart [284].

The next factor associated with angiogenesis is hypoxia-inducible factors (HIFs), including HIF-1, HIF-2 and HIF-3 subtypes; however, that best described in the literature is HIF-1. HIF-1 consist of two subunits: HIF-1α and HIF-1β, which are characterised by a different expression. The expression of *HIF-1β* is continuous and remains steady, whereas the *HIF-1α* expression depends on conditions. Under normoxic conditions, this subunit undergoes degradation. On the other hand, in hypoxic conditions, HIF-1α is hydroxylated, preventing it from being degraded and causing its cell accumulation. HIF-1 regulates the expression of pro-angiogenic factors in hypoxic cells, including VEGF, angiopoietin 1 and 2, platelet-derived growth factor, placental growth factor and MMPs [285,286]. Theodoropoulos’s team confirmed that the high expression of *HIF-1* was associated with the development of high-grade superficial urothelial carcinomas [287]. Moreover, the increased expression of *HIF-1* was positive correlated with disease progression and recurrence and was also associated with poor overall survival [288,289]. The following study demonstrated that the overexpression of *HIF-1α* was also correlated with microvessel density (MVD) [287,288]. Additionally, Theodoropoulos et al. (2005) confirmed that high *HIF-1α* expression was associated with increased proliferative activity and apoptotic rate. Interestingly, the previous analysis showed that P582S and A588T polymorphism in the *HIF-1α* gene causes an increase in transcriptional activity compared to the wild type [287]. Although the study showed that genotypes of studied *HIF-1α* polymorphism did not influence the incidence of transitional cell carcinoma, among patients who underwent radical cystectomy, those with a variant allele (T allele for P582S and A allele for A588T) had significantly worse disease-free survival and disease-specific survival than those without a variant allele [290].

As mentioned above, HIF-1 may induce *MMP* expression. MMPs consist of above 20 proteolytic enzymes involved in tissue remodelling and ECM degradation, allowing cell migration. The proteolytic activity of MMPs may contribute to regulating the activity of enzymes, growth factors, and cytokines. MMPs may cleave proapoptotic factors, resulting in forming apoptosis-resistant cells [291]. On the other hand, MMPs are synthesised as inactive proenzymes, and active MMPs are regulated by tissue inhibitors of metalloproteinases (TIMPs) [292]. The characteristic of selected MMPs are presented as Table 6.

Animal studies were the first to confirm the role of MMPs in the development of bladder cancer. The inhibition of tumour vascularisation, invasiveness and cell proliferation was observed in mice treated with MMP-2 inhibitor (halofuginone) [295]. An analysis of expression level and clinicopathological parameters showed that *MMP-9* expression was higher in deeper tumours than superficial tumours, in invasive tumours compared to in non-invasive tumours and high-grade tumours compared to low-grade tumours. Interestingly, a high expression level of *MMP-9* was accompanied by increased expression of *VEGF* and low expression of anti-angiogenic factor, thrombospondin-1 (*TSP-1*) [295]. Similarly, increased expression of *MMP-2* was observed in muscle-invasive pT2 < or = bladder tumours than in pT1a tumours. Moreover, patients characterised by high expression of *MMP-2* had a worse prognosis than those with low *MMP-2* expression [296]. The next studies confirmed that overexpression of *MMP-9* in tissue was associated with a higher risk of tumour recurrence and poor prognosis [297,298]. Interestingly, despite the lack of differences between the study and control group in the urine level of MMP-7, fourfold elevated MMP-7 concentration in urine was detected in patients with bladder cancer with regional or distant metastasis [299]. Moreover, the BC patients with T2-T4 tumours, G3 higher rates of disease progression and death from bladder cancer were characterised by increased level detected in urine samples [300]. Similarly, other studies confirmed that the activity of MMP-2 might be associated with the tumour stage [301], while mRNA expression of *MMP-9* was evaluated in regard to tumour recurrence [297]. Moreover, MMP-2 protein overexpression in bladder tissue may be an independent prognostic biomarker for BC progression [302]. The meta-analysis confirmed that *MMPs* overexpression might be a prognostic factor predicting poor bladder cancer survival. The increased *MMPs* expression was correlated with poor outcome in bladder cancer [303]. The analysis of twenty SNPs showed that only the *MMP-9* microsatellite ≥24 CA repeat allele and the MMP-12-82 G/G polymorphisms were associated with invasive BC risk [304]. The G/G genotype of *-181A/*G** polymorphism (*rs11568818*) localised in *MMP7* was more common in the BC patients than the control group, but the polymorphism was not correlated with the tumour grade or stage [305]. The 2G/1G+1G/1G genotype of *MMP1* polymorphism (rs1799750) reduced BC risk. Moreover, additional analysis in ‘ever smokers’ and ‘never smokers’ groups showed that the G/1G+1G/1G genotype reduced the risk among ‘ever smokers’ but not in ‘never smokers’ [306]. The -1607 2G/2G of *MMP-1* and −181 G/G genotype of *MMP7* SNP were associated with increased BC risk. Additionally, smoker BC patients were characterised by higher risk for the same SNPs than non-smoker patients in the North Indian population [293]. The 2G/2G genotype of −1607 polymorphism of *MMP-1* increased BC risk in a Turkish with the highest risk in the group of current smokers [294]. The G/G genotype −181A/G polymorphism localised in *MMP-7* gene was associated with a 1.56-fold increased risk of BC compared to the A/A genotype in a Chinese Han population. The SNP may modulate the *MMP-7* expression by affecting the transcriptional activity. Moreover, the A/G or G/G genotype of the studied polymorphism was associated with BC risk in the groups of tumour size ≥ 3 cm. Thus, −181A/G SNP was correlated with the tumour size and tumour node metastasis of BC [307]. 

In addition to pro-angiogenic factors, anti-angiogenic factors, including thrombospondin-1 (TSP-1), may also be associated with BC development. TSP-1 is an extracellular glycoprotein that plays a crucial role in suppressing epithelial cell proliferation, migration and vessel-formation. Moreover, TSP-1, which is secreted by tumour and stromal cells, inhibits MMP-9 and NO/cGMP-related pathways, which results in a limitation in their pro-angiogenic activity. An in vitro study showed that bladder cancer cells were characterised by a decreased level of TSP-1 as compared to normal urothelium cells; moreover, the level of VEGF remained unchanged. Thus, TSP-1 decrease may be the initiation step of angiogenesis in BC [308]. Donmez et al. (2009) found that the lower expression of *TSP-1* was observed in high-grade tumours compared to low-grade tumours [273]. Similarly, the expression was lower in deeper tumours than superficial tumours and invasive tumours than non-invasive tumours. Moreover, the subsequent study showed that the decreased expression level of TSP-1 retained their independent association with disease recurrence and cancer-specific mortality [309,310]. The analysis of −696C/T (rs2664139) polymorphism of *TSP-1* showed that the C/C genotype increased the risk of BC as compared to the C/T and T/T genotypes in the Chinese population. However, the difference was more visible in a group of men. Moreover, the analysis of BC clinical features showed that the studied polymorphism was associated with a higher risk of developing grade III, multiple-tumour and large-BC [311]. On the other hand, in the case of -1223A/G (rs2169830) polymorphism localised in *TSP-1* gene, no significant differences were detected in the genotype frequencies of controls and BC patients in the Chinese population. However, the mRNA expression of the gene in bladder cancer tissues was lower in patients with the A/G genotype than those in those with an A/A genotype, while patients with the G/G genotype were characterised by the lowest expression [312]. In turn, *TSP-2* expression was negatively correlated with T stage, metastasis, grade, cancer cell proliferation and *MMP-9* expression. Therefore, the study suggests that TSP-2 may be involved in malignant aggressiveness and BC progression [313]. 

Fibroblast growth factors (FGFs) play crucial roles in angiogenesis and are involved in embryonic development, tissue regeneration and neoplastic transformation. FGFs consist of above 20 elements that bind four receptors (FGR1-4). However, the most well-studied for angiogenetic activities is aFGF (acid) and bFGF (basic) produced by stromal and endothelial cells. In the extracellular matrix, FGFs form complexes with proteoglycans to avoid degradation. During angiogenesis, FGFs are released by proteinases and then bind to receptors with tyrosine kinase activity. Finally, the started signalling pathways induced proliferation, migration, survival of ECs and increased the expression of other pro-angiogenic factors [314]. Previous studies showed that the overexpression of *bFGF* was positively correlated with muscle invasion, high tumour grade, chemotherapy resistance, high recurrence rate and poor prognosis in BC patients, while *bFGF* mRNA was associated with microvessel density [309,315]. The patients with BC were characterised by an increased serum level of bFGF as compared to the control group [316]. Similarly, the level of bFGF in urine was also elevated in patients with BC, and additionally, the level was correlated with the tumour grade, stage and tumour recurrence [317]. Previous studies showed that *FGFR3* gene located on chromosome 4p16is the most frequent genetic alterations in bladder cancer. The most frequent somatic *FGFR3* mutations are localised in exons 7 (codon 248/249), exon 10, (codon 372) and exon 15 (codon 652); moreover, the mutations were associated with the development of tumours with favourable prognosis (including low-grade non-invasive papillary urothelial cancer and papillary urothelial neoplasms of low malignant potential) [318,319,320,321].

The elements of the angiogenesis pathway listed in the text can be used in diagnostics (Table 7).

## 6. Conclusions

**Figure 4:**
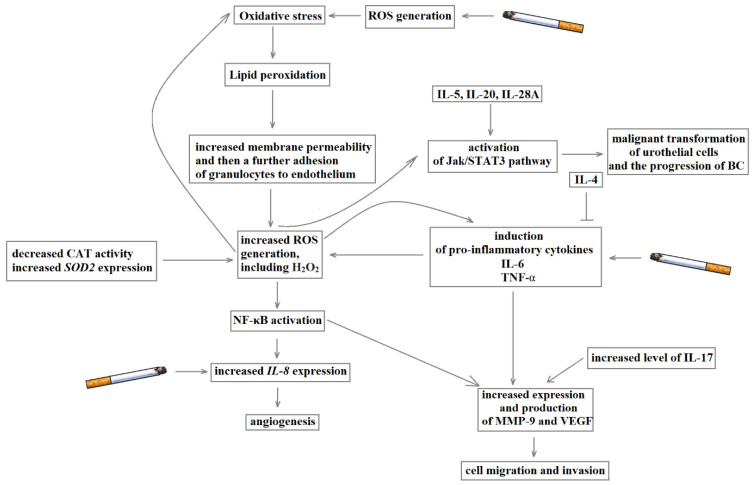
The mutual interaction of oxidative stress, inflammation and angiogenesis in the development of bladder cancer.

In summary, the development of BC is the result of an interaction of disturbances in the course of related biochemical pathways, including oxidative stress, inflammation and angiogenesis. The review allows identifying the common points involved in BC pathomechanism for the analysed methods (Figure 4).

On the one hand, ROS overproduction may induce activity of proinflammatory cytokines, including IL-6 and TNF-α. Thus, oxidative stress is associated with maintaining an inflammatory microenvironment that promotes an increased proliferation of cancer cells. On the other hand, cytokines may further increase ROS production, leading to a vicious circle phenomenon. In angiogenesis, both oxidative stress and inflammation are involved in the activation of pro-angiogenesis factors and the inhibition of anti-angiogenesis compounds. Additionally, the JAK-STAT-3 pathway associated with malignant transformation of urothelial cells and the progression of bladder cancer can be induced by both ROS and proinflammatory cytokines. Therefore, further studies may identify factors involved in the regulation of many related biochemical pathways and reveal potential therapeutic targets.

## Figures and Tables

**Table 1:** Confirmed genetic variants associated with bladder cancer risk in European.

Gene (Protein Product)	Polymorphism	Chromosome Location	Position	Nucleotide Change	SNP Function	Risk Allele (Frequency)	Allelic OR (95%CI)	*p*-Value	Ref.
*AL050403.2*	rs62185668	20p12.2	chr20:10981287	C/A	intron variant	0.22012	1.19 (1.13–1.26)	2 × 10^−11^	[46]
*PSCA, JRK*	rs2294008	8q24.3	chr8:142680513	C/T	5′ UTR variant	0.451005	1.15 (1.10–1.20)	2 × 10^−10^	[47,48]
*TP63, P3H2*	rs710521	3q28	chr3:189928144	T/C	intergenic variant	0.257688	1.19 (1.12–1.27)	6 × 10^−8^	[49]
*CASC11, MYC*	rs9642880	8q24.21	chr8:127705823	G/A G/T	intron variant	0.000000 0.455939	1.21 (1.15–1.28)	7 × 10^−12^	[48]
*AC023421.2, SLC14A1*	rs7238033	18q12.3	chr18:45737001	T/C	intron variant	0.566372	1.2 (1.13–1.28)	9 × 10^−9^	[50]
*TACC3*	rs798766	4p16.3	chr4:1732512	T/C	intron variant	0.795826	1.24 (1.17–1.32)	1 × 10^−11^	[36,51]
*PSD3, NAT2*	rs1495741	8p22	chr8:18415371	G/A	regulatory region variant	0.756073	1.14 (1.09–1.18)	2 × 10^−10^	[52,53]
*UGT1A8, UGT1A10*	rs11892031	2q37.1	chr2:233656637	A/C A/T	intron variant	0.078850, 0.000000	1.19 (1.12–1.27)	1 × 10^−7^	[39,54]
*CCNE1, AC008798.3*	rs8102137	19q12	chr19:29805946	T/C	regulatory region variant	0.306569	1.13 (1.09–1.17)	2 × 10^−11^	[39,55]
*APOBEC3A, AL022318.1*	rs1014971	22q13.1	chr22:38936618	C/T	regulatory region variant	0.628195	1.18 (1.10–1.18)	8 × 10^−12^	[39,53]
*CLPTM1L*	rs401681	5p15.33	chr5:1321972	G/A	intron variant	0.433217	1.12 (1.08–1.16)	4 × 10^−11^	[53,56]
*AC023421.2, SLC14A1*	rs17674580	18q12.3	chr18:45729946	C/A C/T	5′ UTRvariant	0.000000, 0.332221	1.17 (1.11–1.22)	8 × 10^−11^	[56,57]
*LINC02871*	rs6104690	20p12.2	chr20:11007451	G/A G/T	intron variant	0.553042, 0.000000	1.12 (1.08–1.18)	7 × 10^−7^	[53]
*LSP1, TNNI2*	rs907611	11p15.5	Chr11:1852842	G/A	regulatory region variant	0.304845	1.15 (1.09–1.21)	4 × 10^−8^	[53]
*MYNN*	rs10936599	3q26.2	chr3:169774313	C/T	synonymous variant	0.245632	1.18 (1.11–1.23)	5 × 10^−9^	[52,53]
*AL513188.1, CDKAL1*	rs4510656	6p22.3	chr6:20766466	C/A	intron variant	0.38803	1.12 (1.08–1.18)	7 × 10^−7^	[53]
*PAG1*	rs5003154	8q21.13	chr8:81074718	T/C T/G	intron variant	0.51809, 0.00000	1.11 (1.06–1.16)	1 × 10^−6^	[53]
*MCF2L*	rs4907479	13q34	chr13:113004794	G/A G/C	intron variant	0.23256, 0.00000	1.13 (1.07–1.18)	3 × 10^−6^	[53]

**Table 2:** The compounds associated with oxidative stress.

Gene	Enzyme	Gene Location	Characteristics of the Enzyme	References
*COX-2*	prostaglandin-endoperoxide synthase 2 (cyclooxygenase)	1q31.1	is the key enzyme in prostaglandin biosynthesis and acts both as a dioxygenase and as a peroxidase—converts arachidonic acid to prostaglandin endoperoxide H2; COX-2 is naturally inhibited by calcitriol (the active form of Vitamin D)	[58,59,60]
*NOX-4*	NADPH oxidase 4	11q14.3	protects the vasculature against inflammatory stress; NOX-dependent ROS modulation by amino endoperoxides may induce apoptosis in high Nox4-expressing cancer cells	[61]
*iNOS*	inducible nitric oxide synthase	17q11.2.	iNOS produces large quantities of NO upon stimulation, such as by proinflammatory cytokines	[61,62,63,64,65]
*CAT*	catalase	11p13	is a heme enzyme and catalyses the decomposition of hydrogen peroxide to water and oxygen and thereby mitigates the toxic effects of hydrogen peroxide	[66,67,68]
*GPx3*	glutathione peroxidase 3	5q33.1	catalyze the reduction of organic hydroperoxides and hydrogen peroxide (H2O2) by glutathione, and thereby protect cells against oxidative damage; this isozyme is secreted, and is abundantly found in plasma	[69]
*SOD1*	superoxide dismutase 1 [Cu-Zn]	21q22.11	is a soluble cytoplasmic protein, acting as a homodimer to convert naturally occurring but harmful superoxide radicals to molecular oxygen and hydrogen peroxide; this protein also contains an antimicrobial peptide that displays antibacterial, antifungal, and anti-MRSA activity against *E. coli*, *E. faecalis*, *S. aureus*, *S. aureus* MRSA LPV+, *S. agalactiae*, and yeast *C. krusei*	[69]
*SOD2*	manganese-dependent superoxide dismutase (MnSOD)	6q25	transforms toxic superoxide, a by-product of the mitochondrial electron transport chain, into hydrogen peroxide and diatomic oxygen	[69,70]
*PON1*	serum paraoxonase/arylesterase 1	7q21.3	is secreted mainly by the liver; is responsible for hydrolysing organophosphate pesticides and nerve gasses, and mediates enzymatic protection of low-density lipoproteins against oxidative modification	[71,72,73]
*PON2*	serum paraoxonase/arylesterase 2	7q21.3	may act as a cellular antioxidant, protecting cells from oxidative stress—prevents LDL lipid peroxidation, reverses the oxidation of mildly oxidised LDL, and inhibits the ability of MM-LDL to induce monocyte chemotaxis. Hydrolytic activity against acylhomoserine lactones, important bacterial quorum-sensing mediators, suggests the encoded protein may also play a role in defence responses to pathogenic bacteria	[74]

**Table 3:** Potential biomarkers of bladder cancer diagnosis.

Oxidative Stress				
Gene/Protein	Biological Specimens	Molecular Change in Bladder Cancer Course	Comments	References
MDA level	serum	increased		[80]
8-iso-PGF2 α	urine	increased	no correlation was observed between 8-iso-PGF2 α level and the degree of malignancy and invasiveness of BC	[53,82]
*COX-2* expression	bladder cells	increased	*COX-2* expression was inversely correlated with existing of recurrence of NMIBC; high level of *COX-2* expression was associated with an advancing grade and T stage of superficial transitional cell carcinoma	[59,60]
*NOX-4* expression	bladder tissue	overexpression		[61]
NO	bladder tissue, urine and serum	increased		[64,65]
*iNOS* expression	bladder tissue	increased	overexpression of *iNOS* was correlated with a transition to more advanced stages of bladder cancer	[62]
CAT level	serum, blood	reduced		[66]
*CAT* expression and activity	bladder tissue		the low expression of *CAT* may contribute to the recurrence of BC	[67,69,79]
GPx3 activity	plasma	reduced		[69]
GPx1 activity and expression	erythrocytes, leucocytes	increased		[69,98,99]
SOD activity	cell carcinoma, erythrocytes	reduced		[69]
*SOD* expression	cell carcinoma, peripheral blood leucocytes			[83,84,85,86]
SOD level	serum		SOD level in serum and blood was negatively correlated with the stage of bladder cancer—the lowest level of SOD was observed in patients with the most advanced cancer	[66,87]
PON1 concentration	serum	reduced		[71,72]
*PON2* expression	bladder tissue	increased		[74]

**Table 4:** Characteristic of cytokines associated with bladder cancer.

Gene	Name	Location	Characteristic	References
*TNF-α*	tumour necrosis factor alpha	6p21.33	TNF-α is mainly secreted by macrophages and can bind to and thus functions through its receptors TNFRSF1A/TNFR1 and TNFRSF1B/TNFBR. This cytokine is involved in regulating a broad spectrum of biological processes, including cell proliferation, differentiation, apoptosis, lipid metabolism, and coagulation. It has been implicated in various diseases, including autoimmune diseases, insulin resistance, psoriasis, rheumatoid arthritis, ankylosing spondylitis, tuberculosis, autosomal dominant polycystic kidney disease, and cancer.	[128,129,130,131,132,133,134,135,136,137,138,139,140,141,142,143,144,145,146,147,148,149,150]
*IL-4*	interleukin 4	5q31.1	This cytokine is a ligand for the interleukin 4 receptor. This receptor also binds to IL13. IL4 is considered an important cytokine for tissue repair, counterbalancing the effects of proinflammatory type 1 cytokines; however, it also promotes allergic airway inflammation. IL-4 has an essential role in the production of allergen-specific immunoglobin (Ig) E.	[151,152]
*IL-5*	interleukin 5	5q31.1	The cytokine acts as a growth and differentiation factor for both B cells and eosinophils and plays a significant role in regulating eosinophil formation, maturation, recruitment and survival.	[153]
*IL-6*	interleukin 6	7p15.3	The cytokine is involved in inflammation and the maturation of B cells. An elevated level of the encoded protein has been finding in virus infections, including COVID-19.	[154,155,156,157,158,159,160,161,162,163,164,165,166,167,168]
*IL-8*	interleukin 8	4q13.3	IL-8 is a major mediator of the inflammatory response and is secreted by mononuclear macrophages, neutrophils, eosinophils, T lymphocytes, epithelial cells, and fibroblasts. It functions as a chemotactic factor by guiding the neutrophils to the site of infection. This chemokine is also a potent angiogenic factor. The binding of IL-8 to one of its receptors (IL-8RB/CXCR2) increases the permeability of blood vessels, and an elevated level of IL-8 is positively correlated with the greater severity of multiple disease outcomes.	[169,170,171,172,173,174,175,176,177,178,179,180,181,182,183,184]
*IL-17*	interleukin 17	6p12.2	The cytokine is produced by activated T cells. IL-17-mediated downstream pathways induce the production of inflammatory molecules, chemokines, antimicrobial peptides, and remodelling proteins. The cytokine elicits crucial impacts on host defence, cell trafficking, immune modulation, and tissue repair, with a critical role in the induction of innate immune defences. A high level of this cytokine is associated with several chronic inflammatory diseases, including rheumatoid arthritis, psoriasis and multiple sclerosis. The lung damage induced by the severe acute respiratory syndrome coronavirus 2 (SARS-CoV-2) is largely a result of the inflammatory response promoted by cytokines such as IL-17 (cytokine storm).	[185,186,187,188,189,190]
*IL-20*	interleukin 20	1q32.1	The cytokine is part of the larger IL-10 cytokine family. It is produced by keratinocytes and generally functions to enhance innate defence mechanisms. It promotes wound healing by increasing keratinocyte proliferation. IL-20 is considered critical in skin inflammation, and upregulation of its receptor has been shown in patients with psoriasis.	[152]
*IL-28A*	interleukin 28A/interferon lambda 2	19q13.2	*IL-28A* expression can be induced by a viral infection.	[152]
*NF-κB*	nuclear factor kappa B	4q24	NF-κB is a transcription regulator activated by various intra- and extra-cellular stimuli such as cytokines, oxidant-free radicals, ultraviolet irradiation, and bacterial or viral products.	[169,170,191,192,193]
*TGF-β*	transforming growth factor beta 1	19q13.2	The factor regulates cell proliferation, differentiation, and growth and modulates the expression and activation of different growth factors, including interferon-gamma and TNF-α. This gene is frequently upregulated in tumour cells, and mutations in this gene result in Camurati-Engelmann disease.	[194,195,196,197,198,199,200]
*IFN-α*	Interferon alpha	9p21.3	It is produced in response to viral infection as a key part of the innate immune response with potent antiviral, antiproliferative and immunomodulatory properties.	[201,202]
*IFN-β*	Interferon beta	9p21.3	It is released as part of the innate immune response to pathogens.	[201,202]
*IFN-γ*	interferon gamma	12q15	It is secreted by cells of both the innate and adaptive immune systems and binds to the interferon-gamma receptor, which triggers a cellular response to viral and microbial infections	[201,202]

**Table 5:** The elements of the inflammation involved in bladder cancer development.

Inflammation				
Gene/Protein	Biological Specimens	Molecular Change in Bladder Cancer Course	Comments	References
*NF-κB* expression	bladder tissue	increased	NF-κB expression is associated with histologic grade and T category in bladder urothelial cancer	[191]
*IL-8* expression	bladder tissue	increased	*IL-8* expression may be associated with the metastatic potential of human transitional cell carcinoma	[171]
TNF-α level	urine	increased	the enzyme level is associated with bladder cancer progression	[135,136]
IL-6 level	serum	increased	high serum level of IL-6 was associated with metastasis and poor prognosis	[155]
*IL-17* expression	peripheral blood	increased	worse prognosis may be correlated with high expression of *IL-17*	[187,188]

**Table 6:** Characteristics of MMPs associated with BC development.

Gene	Gene Localised	Name (Aliases)	Location	Substrates	Activation Pathway	References
*MMP-1*	11q22.2	interstitial collagenase (CLG, CLGN)	secreted	collagens: I, II, III, VII, VIII, X, gelatin	The plasmin has been described and other serine proteases, i.e., kallikrein, trypsin, neutrophil elastase, cathepsin G, tryptase and chymase may be involved in the activation of proMMP-1.	[293,294]
*MMP-2*	16q12.2	gelatinase-A, 72 kDa gelatinase	secreted	gelatin, collagens: I, II, III, IV, Vii, X	A complex of membrane-type 1 MMP (MT1-MMP/MMP14) and tissue inhibitor of MMP-2 recruits pro-MMP 2 from the extracellular milieu to the cell surface. Activation then requires an active molecule of MT1-MMP and autocatalytic cleavage. Clustering of integrin chains promotes MMP-2 activation. Another factor that will support the activation of MMP-2 is cell-cell clustering. A wild-type activated leukocyte cell adhesion molecule (ALCAM) is also required to activate the MMP-2.	[146,147,293,295,296,297,298,299,300,301,302,303,304,305,306]
*MMP-7*	11q22.2	matrilysin, PUMP 1 (MMP-7, MPSL-1, PUMP-1)	secreted	fibronectin, laminin, collagen IV, gelatin	Pro-MMP7 is converted from the latent form to the active form by endoproteinases and plasmin. Plasmin cleaves at the site recognisable to trypsin is considered as the possible physiological activator.	[299,307]
*MMP-9*	20q13.12	gelatinase-B, 92 kDa gelatinase (CLG4B, GELB, MANDP2, MMP-9)	secreted	gelatin, collagen IV, V	The proMMP-9 includes a cysteine residue in the N-terminal pro-domain that binds to the zinc atom in the active site thus maintaining latency. Activation of MMP-9 requires a disruption of the cysteine interaction with the zinc atom. MMP-9 activators include MMP-2, MMP-3, MMP-7, MMP-10, MMP-13, cathepsin G and urokinase/plasmin.	[296,297,298,299,304]
*MMP12*	11q22.2	macrophage metalloelastase (HME, ME, MME, MMP-12)	secreted	elastin, fibronectin, collagen IV	Neutrophil elastase may be required for the proteolytic activation of pro-MMP-12	[304]

**Table 7:** The factors of the angiogenesis pathway involved in angiogenesis.

Angiogenesis				
Gene/Protein	Biological Specimens	Molecular Change in Bladder Cancer Course	Comments	References
*VEGF* expression	bladder tissue	increased	The VEGF-A level in tissue was correlated with BC grade; *VEGF* expression was higher in deeper tumours than superficial tumours and invasive tumours compared to non-invasive tumours.	[272,275]
*VEGFR-1* expression	bladder tissue	increased	mRNA level of *VEGFR* was correlated to the pathologic stage of BC.	[275]
*HIF-1* expression	bladder tissue	increased	The increased *HIF-1* expression was positive correlated with disease progression and recurrence and was also associated with poor overall survival.	[288,289]
*MMP-9* expression	bladder tissue	increased	*MMP-9* expression was higher in deeper tumours compared to superficial tumours, in invasive tumours compared to non-invasive tumours and high-grade tumours than in low-grade tumours; overexpression of *MMP-9* in tissue was associated with a higher risk of tumour recurrence and poor prognosis.	[295,297,298]
MMP-2 level	bladder tissue	increased		[301,302]
TSP-1 level	bladder cells (in vitro study)	reduced		[308]
*TSP-1* expression	bladder tissue		The lower expression of *TSP-1* was observed in high-grade tumours than in low-grade tumours.	[309,310]
*bFGF* expression	bladder tissue	increased	The overexpression of *bFGF* was positively correlated with muscle invasion, high tumour grade, chemotherapy resistance, high recurrence rate and poor prognosis in BC patients.	[309,315]
bFGF level	urine, serum			[316,317]
